# INCOMPLETE ADVANCE CARE PLANNING? CORRELATES OF PLANNING WITHOUT PERSONAL CONVERSATIONS

**DOI:** 10.1093/geroni/igz038.1589

**Published:** 2019-11-08

**Authors:** Kathrin Boerner, Deborah Carr, Katherine M Ornstein, Sara Moorman

**Affiliations:** 1 University of Massachusetts Boston, Boston, Massachusetts, United States; 2 Boston University, Boston, Massachusetts, United States; 3 Icahn School of Medicine at Mount Sinai, New York, New York, United States; 4 Boston College, Chestnut Hill, Massachusetts, United States

## Abstract

In the course of advance care planning (ACP), people may elect any of the following: a living will, a durable power of attorney for health care, and discussions with family members and health care providers. A small proportion of planners complete legal documents without discussing them with others (formal planning only, FPO). If people who have done FPO become incapacitated, their family and health care professionals may lack guidance on how to direct their care. To better understand this group, we drew on four large surveys of community-dwelling adults. Social isolation, measured by living alone and lack of a confidante, increased the odds of FPO across all studies. We also found some evidence that economic disadvantage and depressive symptoms were linked with FPO. We discuss implications for policy and practice, underscoring that ACP is yet another important domain affected by the crisis of social isolation in old age.

